# Effect of Transition Layers on the Microstructure and Properties of CMT Additively Manufactured Steel/Copper Specimens

**DOI:** 10.3390/ma18081734

**Published:** 2025-04-10

**Authors:** Xuyang Guo, Yulang Xu, Jingyong Li, Cheng Zhang

**Affiliations:** 1 Advanced Welding Technology Provincial Key Laboratory, Jiangsu University of Science and Technology, Zhenjiang 212003, China; zxdx2019@163.com (X.G.);; 2 School of Naval Architecture and Intelligent Manufacturing, Jiangsu Maritime Institute, Nanjing 211199, China

**Keywords:** steel/copper CMT additive manufacture, transition layer, microstructure observation, mechanical properties, elemental diffusion

## Abstract

During the cold metal transfer (CMT) arc additive manufacturing process of steel/copper bimetallic materials, interfacial penetration cracks have been observed due to the significant differences in thermal and physical properties between steel and copper. To mitigate the occurrence of these penetration cracks and enhance the interfacial elemental diffusion at the steel/copper junction, this study aims to fabricate high-performance steel/copper bimetallic materials with a uniform microstructure using CMT arc additive manufacturing techniques. A reciprocating additive sequence was adopted, with steel deposited first, followed by copper. Four different interlayer compositions, Cu-Ni, Fe-Ni, Cu-Cr, and Ni-Cr, were applied to the steel surface before the deposition of aluminum bronze. These interlayers served as a transition between the steel and copper materials. The manufacturing process then continued with the deposition of aluminum bronze to achieve the desired bimetallic structure. After the addition of interlayers, all four sets of samples exhibited excellent macroscopic formability, with clear and smooth interlayer contours and no visible cracks or collapse defects at the junction interfaces. The mechanical properties of the composite walls were enhanced following the addition of the interlayers, with an increase in tensile strength observed across the samples. The sample with the Fe-Ni interlayer showed the most significant improvement, with a 52% increase in impact energy absorption. Furthermore, the sample with the Fe-Ni interlayer demonstrated a higher average hardness level than the other groups, which was associated with the distribution and content of the iron-rich phase and the β′ phase.

## 1. Introduction

Copper and its alloys are extensively used in heat dissipation components across the aerospace, nuclear power, and electronic communications industries because of their outstanding properties, such as tensile strength, corrosion resistance, and ductility [1,2]. However, the high cost and relatively low strength of pure copper have motivated the exploration of copper/steel bimetallic composites, which combine the beneficial properties of both materials to achieve enhanced performance and cost-effectiveness [3].

The considerable disparity in the physical properties of copper and steel, along with their poor weldability, poses significant challenges for the fabrication of high-performance composite materials. Traditional fabrication techniques, such as composite rolling [4,5,6], spray deposition [7,8,9,10], powder sintering [11,12], and various welding methods [13,14,15,16,17], are commonly employed. In contrast, additive manufacturing (AM) offers distinct advantages, including mold-free production, greater design flexibility, and shortened production cycles, making it a promising approach for fabricating complex bimetallic structures [17,18,19].

Recent studies on AM-based copper/steel composites have primarily focused on process optimization, such as process parameters [20,21,22], scanning strategies [23,24] and interface characterization [25,26,27]. Researchers have also investigated the use of transition layer fillers to improve interfacial properties. Zhang [28] developed a transition welding wire for copper/steel gradient connections. By incorporating a Cu-Ni interlayer welding wire, the continuous diffusion of Fe, Ni, and Cu elements into the low-alloy steel and silicon bronze matrix was enhanced, improving the heterogeneity of Fe-rich phases in the silicon bronze matrix and achieving a more uniform microhardness distribution in the transition zone. Liu et al. [29] performed magnetic-field-assisted TIG welding on T2 red copper/Q235 steel dissimilar materials using HS201 pure copper welding wire, achieving a joint tensile strength of 223.5 MPa and fusion zone microhardness of 659 HV0.2. Both methods enabled the gradient connection of copper/steel bimetals with improved performance. Zhang [30] successfully fabricated a bimetallic structure of QCr0.8 high-strength, high-conductivity copper alloy and 06Cr13Ni4Mo (S06) stainless steel using laser powder hybrid additive manufacturing (LPH-AM) technology. By employing In718 alloy as an interlayer and optimizing the process parameters, a relative density of 99.9% was achieved, along with excellent metallurgical bonding. The vertically assembled specimens demonstrated an average tensile strength of 300.3 ± 10.6 MPa and a fracture elongation of 15.0 ± 1.4%.

Building upon previous studies, laser-based AM utilizes powder to fill transition layers, and the filler material for transition layers in arc-based AM is primarily introduced via flux-cored arc welding (FCAW). This paper presents a novel method for fabricating composite metals by filling transition layers with four different powders (Cu-Ni, Fe-Ni, Cu-Cr, Ni-Cr) in cold metal transfer wire arc additive manufacturing (CMT-WAAM) so as to enhance interfacial integrity and optimize mechanical properties. We aimed to validate the feasibility of incorporating transition layers at the interface between the two materials, investigate the microstructure and phase changes of composite metal samples after the transition layer was filled, and explore the influence of different transition layers on tested mechanical properties.

## 2. Experimental Procedures

### 2.1. Testing Materials

The base material utilized was a Q235 steel plate with dimensions of 200 mm × 80 mm × 20 mm (length × width × thickness). The deposition materials consisted of 1.2 mm diameter S216 aluminum bronze welding wire and ER120S-G high-strength steel welding wire. The chemical compositions of both the welding wires and the base material are presented in Table 1.

This study systematically designed four transition layer powder systems (Cu-Ni, Fe-Ni, Cu-Cr, Ni-Cr) to optimize the interface of metal matrix composites. The Ni element can form unlimited mutual solubility with Cu and Fe, thereby enhancing the solubility of Cu. The Cr element significantly improves the corrosion resistance and hardness of aluminum bronze alloy. The final transition powder components and their proportions used in this experiment, after optimizing the composition ratio, are shown in Table 2. All metal powders were homogenized using a single-vessel planetary high-energy ball mill (Pulverisette 6, Fritsch GmbH, Idar-Oberstein, Germany) at a rotational speed of 180 rpm for 10 min. For preparation of the transition layer, polyvinyl alcohol (PVA, Sigma-Aldrich, St. Louis, MO, USA) dissolved in anhydrous ethanol (mass ratio 1:5) was employed as the binder. The mixture was uniformly applied onto the cladding layer using a brush, followed by thermal curing at 75 °C for 10 min via a hot air gun to ensure complete solidification prior to the additive manufacturing process.

### 2.2. Testing Equipment

The testing equipment included optical microscopes (SZ61 and BX51M, Olympus Co., Ltd., Tokyo, Japan), an X-ray diffraction device (SmartLab9kW, Rigaku Co., Ltd., Tokyo, Japan), and EDS/EBSD (S3400N, Hitachi Co., Ltd., Tokyo, Japan), and a direct-reading spectrometer (MAXX LMM05, Spectro Co., Kleve, Germany). Both EDS and EBSD were independent accessory modules coupled with the SEM, achieving spatial data matching through the same electron beam path. Since TEM was not utilized in this study, no TEM-related EDS configuration was involved. The microstructure observation samples for this experiment were prepared with dimensions of 10 mm × 10 mm × 5 mm, cut along the rolling direction, and sequentially polished using abrasive papers of 400#, 800#, 1200#, 1500#, and 2000#. Subsequently, diamond polishing agents with particle sizes of 2.5, 1.5, and 0.5 were applied for mechanical polishing. After polishing, a ferric chloride hydrochloric acid solution (Metallographic Equipment Co., Ltd., Jiangxi, China) was prepared by mixing 5 g of FeCl_3_, 5 mL of HCl, and 50 mL of C_2_H_5_OH for the Cu corrosion experiment, with a 4% nitrate alcohol solution (Metallographic Equipment Co., Ltd., Jiangxi, China) for the Fe corrosion experiment.

Simultaneously, mechanical property evaluations were conducted, including Vickers hardness (KB30S, KB Co., Ltd., Assenheim, Germany), tensile testing (CMT5205, MTS, Minneapolis, MN, USA), and impact testing (SANS, ZBC2302D, Metes Industrial Systems Co., Ltd., Shanghai, China). Hardness measurements were taken at 2 mm intervals under a 0.1 kg load for 15 s, with 300 points tested for each specimen and averaged to obtain the final results. Tensile tests were carried out at a rate of 1 mm/min at room temperature, with three specimens tested per group, and the values were averaged for the final results.

## 3. Results and Discussions

### 3.1. Microstructure Evolution Analysis

**Figure 1:**
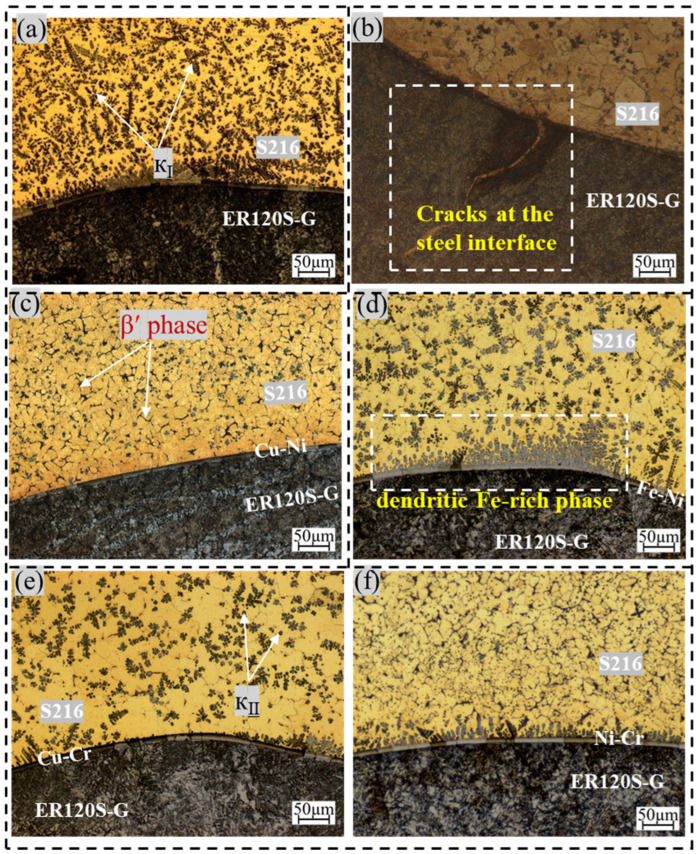
Microstructure observation with and without transition layer: (**a**) without transition layer, (**b**) crack, (**c**) Cu-Ni, (**d**) Fe-Ni, (**e**) Cu-Cr, (**f**) Ni-Cr.

Figure 1a shows a considerable presence of large, flower-shaped iron-rich precipitates (κ_Ⅰ_) and spherical iron-rich phases (κ_Ⅱ_) dispersed near the copper side of the interface [29]. As the molten pool solidifies, the temperature gradient undergoes a shift, causing the iron-rich phases to change shape; these phases evolve from their initial spherical and flower-like forms into dendritic structures, ultimately adopting a fully dendritic morphology once cooling is complete. Cracks at the steel interface, resulting from copper infiltration, are evident in Figure 1b.

Following the filling of the transition layer, as depicted in Figure 1c–f, the interface region primarily consisted of the iron-rich phase, β phase, and α-Cu phase. The fusion line at the interface was clearly delineated. In comparison to samples without the transition layer, both the size and quantity of the iron-rich phases on the copper side were reduced. This observation suggests that the transition layer filling effectively hindered the diffusion of Fe elements, thereby lowering the Fe content at the interface. As shown in Figure 1d, an excessive amount of Fe results in the formation of numerous epitaxially grown iron-rich dendrites at the interface. A comprehensive comparison indicates that Ni plays a significant role in inhibiting the diffusion of Fe elements, as specimens containing Ni exhibited fewer and smaller iron-rich phases.

### 3.2. Phase Composition Analysis

#### 3.2.1. XRD Analysis

**Figure 2:**
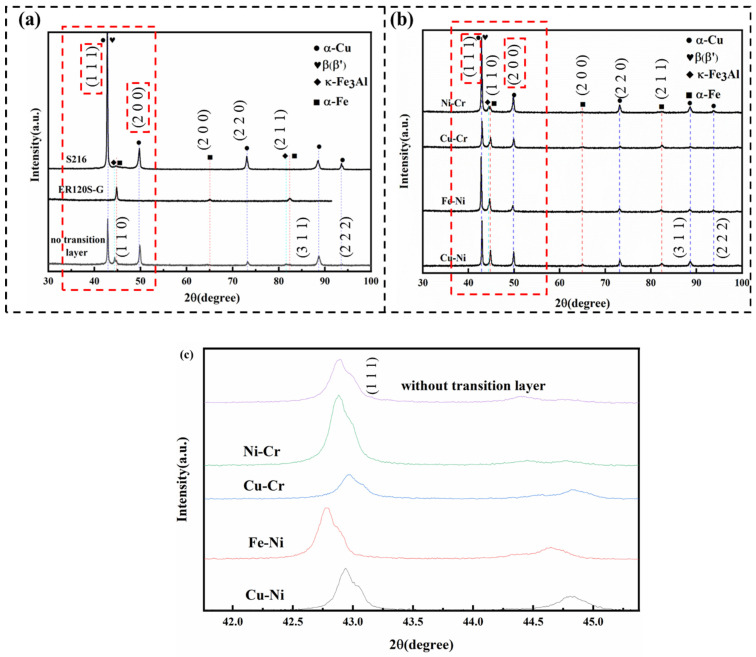
XRD diffraction patterns: (**a**) ER120S-G, S216, and without transition layer; (**b**) with different transition layers; (**c**) partial enlarged data.

As shown in Figure 2a,b, the room temperature microstructure of the aluminum bronze cladding metal predominantly consisted of the α-Cu, β′, and κ-Fe_3_Al phases, while the high-strength steel cladding metal was primarily composed of the α-Fe phase. The XRD patterns before and after filling the transition layer displayed no noticeable change in diffraction peak types, indicating that no new phases formed in any of the four specimen groups. This suggests that the transition layer did not influence the phase composition of the composite thin-walled specimens.

The XRD patterns of the composite metal walls revealed distinct diffraction peaks corresponding to the (111) and (200) crystal planes. During the steel/copper welding process, the diffusion of Fe elements triggers the precipitation of the β-phase and the formation of a gradient (Cu, Fe)_3_Al-type β′ phase, which leads to a decrease in the intensity of the (111) diffraction peak compared with S216 aluminum bronze. As illustrated in Figure 2c, a comparison of diffraction intensities among specimens with different transition layers showed that the (111) peak intensity in specimens with Ni-Cr and Fe-Ni transition layers was substantially higher than in those without any transition layer.

Metallographic analysis further indicates that the Ni-Cr and Fe-Ni transition layers moderately inhibited the diffusion of Fe and Cu, resulting in an increase in the β′ phase at the interface compared with the other samples and a higher concentration of the κ_Ⅰ_ intermetallic compound phase. The lattice compatibility between these phases and the matrix likely enhanced the diffraction response of the (111) crystal plane. In contrast, specimens with other transition layers exhibited simpler interfacial phase compositions and a more dispersed distribution of precipitated phases, which led to a reduction in the intensity of the characteristic diffraction peaks.

#### 3.2.2. EDS Analysis

**Figure 3:**
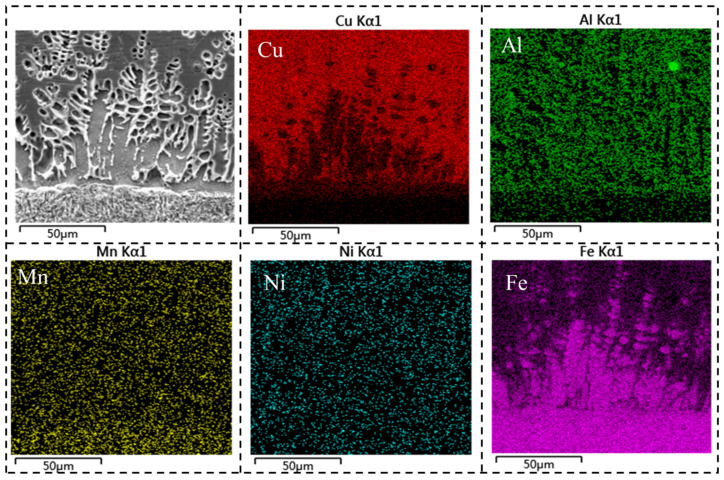
Results of EDS surface energy spectrum scanning.

Figure 3 illustrates the microstructural analysis of aluminum bronze, which revealed three distinct distribution patterns of iron-rich phases. The first pattern consisted of spherical iron-rich phases, which were evenly distributed throughout the aluminum bronze matrix. The second pattern was characterized by flower-like iron-rich phases, which appeared randomly within the silicon bronze matrix. The third pattern showed iron-rich phases aligned along the interface of the transition layer. Additionally, the concentration of iron-rich phases in aluminum bronze was notably lower than that in the transition layer, primarily because of the increased diffusion distance. As the diffusion distance increased, the iron content diffusing from the low-alloy steel deposition layer gradually diminished, resulting in a lower concentration of iron-rich phases in the aluminum bronze.

In this region, the Cu element existed predominantly as a copper-rich matrix, while the aggregated structure at the interface consisted mainly of Fe with a small amount of Al. It can be inferred that the iron-rich phase at the interface was composed of Fe_x_Al dendrites. Given that high-strength steel has a higher solidification point than aluminum bronze, the high-strength steel cladding solidified before the aluminum bronze. During solidification, iron-rich columnar dendrites form and grow along the deposition direction of the AM process into the molten aluminum bronze under the influence of temperature gradients. Simultaneously, driven by the chemical potential gradient between the aluminum bronze matrix and the high-strength steel matrix, Al diffuses intensely from the aluminum bronze matrix into the iron-rich columnar dendritic regions, leading to the enrichment of Al in these areas.

**Figure 4:**
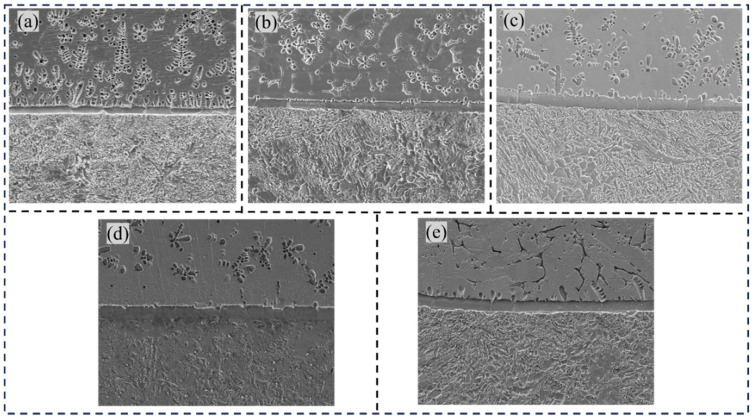
Electron scanning morphology: (**a**) without transition layer, (**b**) Cu-Ni, (**c**) Fe-Ni, (**d**) Cu-Cr, (**e**) Ni-Cr.

**Figure 5:**
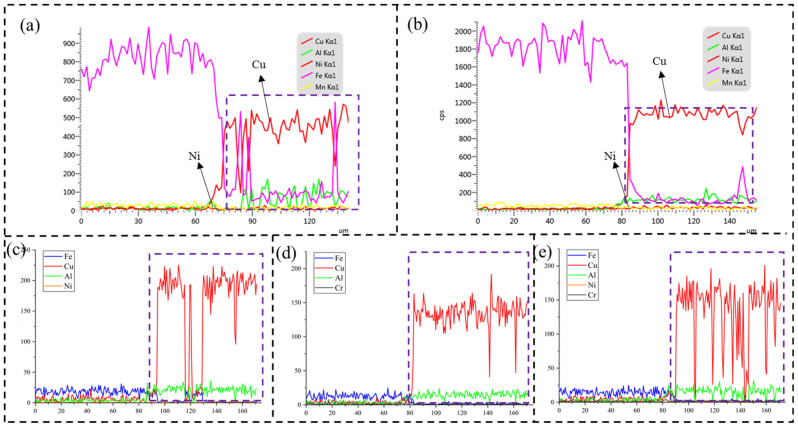
EDS line scanning: (**a**) without transition layer, (**b**) Cu-Ni, (**c**) Fe-Ni, (**d**) Cu-Cr, (**e**) Ni-Cr.

Figure 4a illustrates the bonding interface without the transition layer. A significant population of dendritic Fe-rich phases was observed at the interface region, with analogous dendritic Fe-rich phases also detected within the aluminum bronze matrix. As shown in Figure 5a, the elemental interdiffusion was generally restricted, with mutual diffusion between Fe and Cu occurring predominantly at the interface regions. Figure 4b illustrates the bonding interface after the application of a Cu-Ni transition layer. The fusion line between the steel and the transition layer appeared smooth and straight, with a reduced iron-rich phase at the interface. As shown in Figure 5b, the iron (Fe) content gradually decreased, while the copper (Cu) and nickel (Ni) contents increased progressively. This trend corresponded to the composition of the transition layer filler material (Cu-Ni welding wire), with negligible diffusion between Fe and Cu at the interface. Figure 4c displays the bonding interface after filling with an Fe-Ni transition layer. The iron-rich phase at the interface was present in the form of dendritic crystals. As observed in Figure 5c, the elemental distribution at the interface showed abrupt changes, indicating that the transition layer consisted primarily of Fe-Ni compounds. With increasing diffusion distance, the diffusion of Cu into the low-alloy steel substrate decreased sharply, reducing the likelihood of penetration cracks on the steel side. Figure 4d shows the bonding interface after filling with a Cu-Cr transition layer, where the transition layer remained dominated by a copper-rich matrix and iron-rich phases. As observed in Figure 5d, elemental diffusion was essentially absent at the interface, with the population of Fe-rich phases in these regions remaining relatively sparse. Figure 4e presents the bonding interface with an Ni-Cr transition layer. A distinct steel melting zone is observed at the fusion line. As shown in Figure 5e, alternating variations of Fe and Cu elements were detected at the interface. In conclusion, all four transition materials (Cu-Ni, Fe-Ni, Cu-Cr, and Ni-Cr) achieved excellent metallurgical bonding with low-alloy steel. After the transition layer was added, the fusion line at the interface became smooth and straight. In the absence of the transition layer, mutual diffusion of Cu and Fe elements occurs at the interface. However, with the transition layer, the interdiffusion of Cu and Fe at the interface is significantly reduced, which helps prevent the formation of penetration cracks. On the aluminum bronze side, fluctuations in copper content were observed as the scanning path traversed iron-rich phases on the copper side, with decreases in copper content consistently accompanied by increases in iron content. This phenomenon was particularly noticeable in the Ni-Cr transition layer specimen, where numerous fine iron-rich κ_Ⅱ_ phases were dispersed near the copper-side interface, further corroborating the line scanning results.

#### 3.2.3. EBSD Analysis

**Figure 6:**
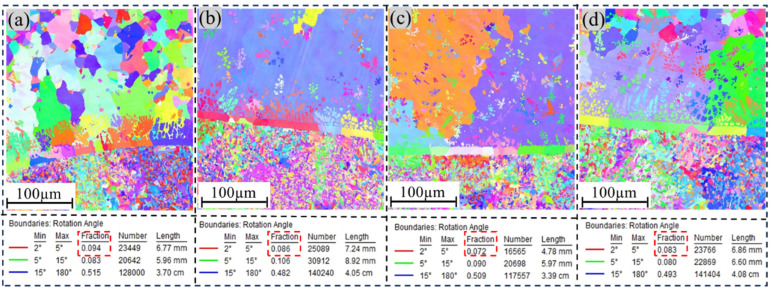
EBSD IPF diagram analysis; (**a**) Cu-Ni, (**b**) Fe-Ni, (**c**) Cu-Cr, (**d**) Ni-Cr.

An in-depth analysis of grain orientation at the interface between the transition layer and aluminum bronze was performed using electron backscatter diffraction (EBSD) technology. As shown in Figure 6, the study revealed that the interfacial region consisted primarily of a Cu-rich matrix (α-Cu grains) and columnar Fe-rich grains formed through diffusion. At the interface, the copper layer exhibited relatively smaller grain sizes, which could be attributed to the high thermal conductivity of the initial copper layers (layer1 and layer2). This enhanced thermal conductivity significantly increased the cooling rate of the molten pool, promoting grain refinement. However, along the build direction, the copper layer demonstrated a gradual coarsening of grain sizes. This phenomenon arose from the significantly lower thermal conductivity of the underlying steel substrate compared with the copper layer. As the number of deposited layers increased, the cooling rate decreased, leading to grain coarsening. Concurrently, Fe grains near the interface exhibited a columnar morphology, with their size and density progressively decreasing as the distance from the interface increased. At the interface, low-angle grain boundaries (LAGBs) dominated the structure. In the Cu-Ni specimen, the proportion of LAGBs was measured to reach 9.4%. The Cu-Cr specimen exhibited a higher proportion of LAGBs than the other specimens. This suggests that the addition of Ni contributed to grain refinement at the interface. The increased proportion of LAGBs, characterized by lower energy and stronger bonding capacity, positively contributed to enhancing the plasticity and toughness of the material.

**Figure 7:**
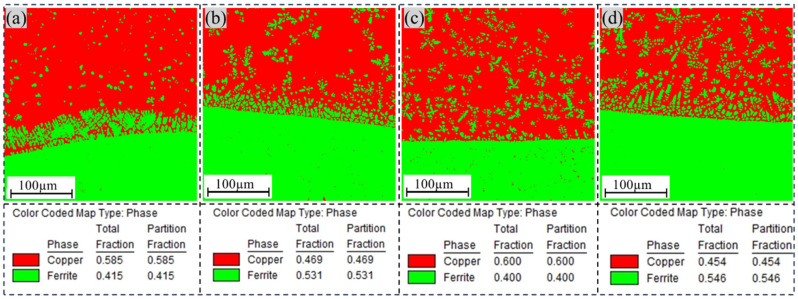
EBSD phase diagram analysis; (**a**) Cu-Ni, (**b**) Fe-Ni, (**c**) Cu-Cr, (**d**) Ni-Cr.

As shown in Figure 7, a comparative analysis of the phase distribution diagrams for different transition-layer samples revealed notable variations in both the content and distribution of the iron-rich phase. Specifically, the sample with a Cu-Cr transition layer contained only 40% iron-rich phase, which was uniformly distributed within the Cu matrix. In contrast, the sample with a Ni-Cr transition layer exhibited a significantly higher proportion of 54.6% iron-rich phase. This discrepancy can likely be attributed to the increased inhibitory effect of the Cu-Cr intermediate layer on the diffusion of iron (Fe) elements. Additionally, in the Cu-Ni sample, the iron-rich phase was located primarily at the interface between the two metals, with a small and uniform size observed in regions away from the boundary.

### 3.3. Comparative Analysis of Mechanical Properties

#### 3.3.1. Comparison of Hardness

**Figure 8:**
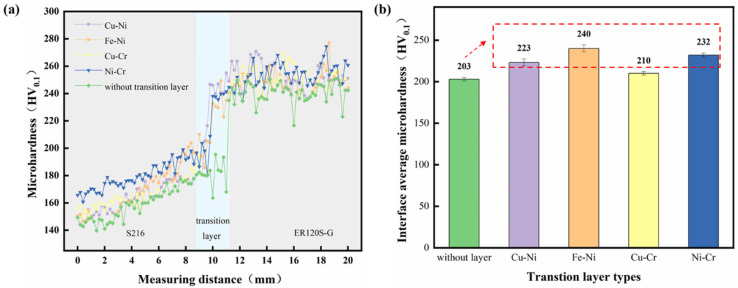
(**a**) Results of microhardness test; (**b**) interface average microhardness.

As shown in Figure 8a, the microhardness analysis revealed average values of 150–160 HV_0.1_ for the copper side and 240–250 HV_0.1_ for the steel side, demonstrating a gradual hardness transition from the aluminum bronze to the high-strength steel interface. Following the application of the transition layer, both the aluminum bronze and high-strength steel sides exhibited slight hardness enhancements compared with specimens without the transition layer. This trend in hardness distribution was linked to the precipitation of the κ phase, as confirmed by metallographic analysis. In the region near the interface, the Fe content diffused from the steel side to the copper side increases significantly. During the cooling process, the solubility of Fe decreases, leading to a higher quantity and denser distribution of precipitated Fe-rich phases. These Fe-rich phases provide a second-phase strengthening effect on the copper-rich matrix, resulting in the observed hardening trend. The average hardness at the interface of the five groups of samples is shown in Figure 8b. For specimens with Ni-Cr and Fe-Ni transition layers, the hardness near the aluminum bronze interface was significantly higher compared with the other specimen groups. The Ni-Cr transition layer sample demonstrated a hardness enhancement of 29 HV_0.1_ compared with the sample without the transition layer, representing a significant increase of approximately 14.3%. The Fe-Ni transition layer specimen exhibited a greater hardness improvement of 37 HV_0.1_, corresponding to a notable 18.2% rise. The increased hardness in the Ni-Cr transition layer specimen was attributed to the numerous finely dispersed κ_II_ precipitates within the aluminum bronze matrix, which induced a pronounced second-phase strengthening effect. In the Fe-Ni transition layer specimen, the elevated Fe content in the transition zone, along with the presence of large Fe-rich phase constituents, collectively contributed to the higher hardness observed throughout the transition region. The Fe-rich phases exerted a secondary phase strengthening effect on the matrix, leading to enhanced average microhardness at the specimen interface after the introduction of transition layers. Notably, the divergent suppression efficacy of Fe elemental precipitation across different transition layers resulted in variations in the spatial distribution and population density of Fe-rich phases, thereby contributing to measurable differences in hardness profiles.

#### 3.3.2. Comparison of Tensile Properties

**Figure 9:**
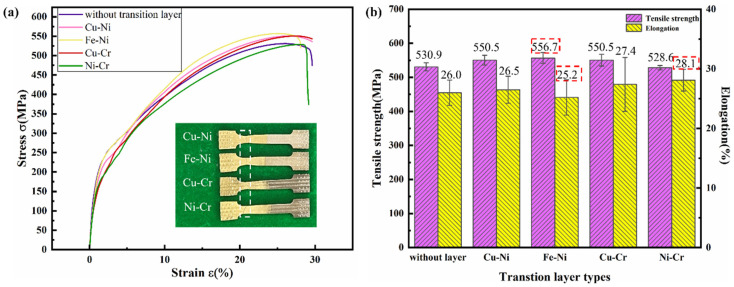
(**a**) Tensile stress–strain curve; (**b**) tensile strength and elongation of thin wall specimens.

Figure 9a presents the tensile stress–strain curves of the specimens, while the detailed tensile test results are shown in Figure 9b. In Figure 9b, the error bars indicate the standard deviation derived from triplicate experiments, demonstrating the reproducibility of the data. All specimens with Ni-containing transition layers exhibited superior tensile strength to that of the sample without the transition layer, demonstrating the effectiveness of Ni-based interlayers in enhancing interfacial bonding. Notably, the Fe-Ni transition layer specimen achieved an average tensile strength of 556.7 MPa, representing a significant 25.8 MPa enhancement compared with the sample without the transition layer. Fractures occurred in the top region of S216 aluminum bronze deposition layers across all specimen groups. This was because the location was far from the transition layer, which significantly reduced the iron content diffused there, resulting in a decrease in the iron-rich phase in the copper matrix and a weakening of the strengthening effect on the matrix. As shown in Figure 9b, the Ni-Cr transition layer specimen demonstrated a postfracture elongation of 28.1% compared with other transition layer samples and showed an 8.1% improvement over the sample without the transition layer. In contrast, the Fe-Ni transition layer sample exhibited reduced ductility, with a postfracture elongation of only 25.2%, representing a 3.1% decrease compared with the sample without the transition layer. This divergence can be attributed to the dual effects of iron-rich phases: while they enhance the tensile strength of the aluminum bronze matrix through precipitation hardening, their inherent brittleness simultaneously compromises the material′s plasticity, ultimately diminishing its elongation capacity. The longer error bars in the postfracture elongation of the Cu-Cr transition layer samples indicate potential instability in the influence of this transition layer on the material′s ductility.

#### 3.3.3. Comparison of Impact Properties

**Figure 10:**
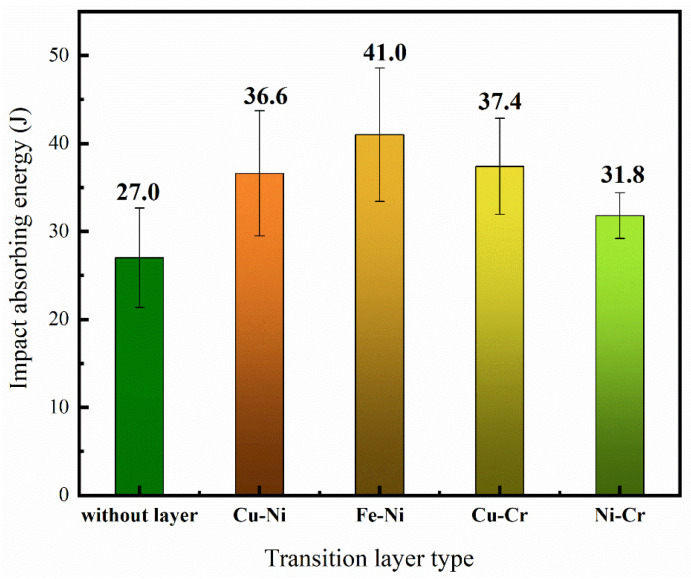
Results of impact test.

As shown in Figure 10, the impact-absorbed energy of the four groups of thin-walled specimens with transition layers was improved compared with the specimen without a transition layer, indicating enhanced interfacial bonding strength between the steel and copper after the addition of transition layers. Among these, the specimen with the Fe-Ni transition layer showed the most significant improvement, with an increase of 14 J, while the Ni-Cr transition layer specimen exhibited the smallest enhancement, with an increase of only 4.8 J in impact-absorbed energy. This difference may be attributed to the higher concentration of the β′ phase in the interfacial region of the Ni-Cr transition layer specimen. Although the β′ phase has high hardness, its lower ductility and toughness may have contributed to the reduced overall impact toughness of the specimen.

**Figure 11:**
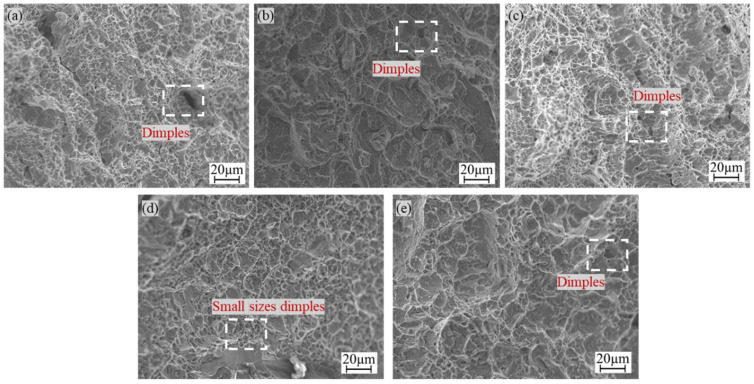
Impact fractures with different transition layers: (**a**) without transition layer, (**b**) Cu-Ni, (**c**) Fe-Ni, (**d**) Cu-Cr, (**e**) Ni-Cr.

Overall, Figure 11 shows that dimples were present in the fracture morphology of all five groups of specimens, along with “quasicleavage” facets and tear ridges, indicating quasicleavage fracture characteristics. As shown in Figure 11c, the Fe-Ni transition layer specimen exhibited larger, deeper, and more uniformly distributed dimples than the other three groups of specimens, resulting in higher impact absorption energy. In contrast, the Cu-Ni, Cu-Cr, and Ni-Cr transition layer specimens showed relatively fewer and smaller dimples, leading to weaker impact absorption energy and lower impact toughness. In conclusion, the Fe-Ni transition layer specimen demonstrated the highest impact absorption energy, which further supports the results of the impact tests.

## 4. Conclusions

The article successfully fabricated steel/copper composite metal samples by incorporating a transition layer. The research findings demonstrated:

(1) In the absence of a transition layer in the sample, flower-shaped iron-rich precipitates (κ_Ⅰ_) and spherical iron-rich phases (κ_Ⅱ_) were dispersed near the copper side of the interface, while cracks formed at the steel/copper interface. After the transition layer was introduced, the fusion line at the interface was clearly delineated, and the Fe-enriched phase, β phase, and α-Cu phase were more evenly distributed. The number of iron-rich phases at the copper/steel interface decreased as the distance from the interface increased, effectively suppressing the diffusion of iron and reducing interfacial penetration cracks. Specifically, the Ni-Cr and Fe-Ni transition layers demonstrated a weaker suppression of iron diffusion, resulting in a higher concentration of iron-rich phase compounds at the interface.
(2) The phase composition of the four samples showed no significant changes after the transition layer was added, consisting primarily of the α-Cu phase, β′ phase, and κFe_3_Al phase. EDS analysis revealed that the addition of the transition layer altered the element distribution at the interface. The transition layer promoted the interdiffusion of Fe, Ni, Cu and other elements.
(3) Hardness tests revealed a gradual increase in hardness from the copper side to the steel side across all samples, with the Fe-Ni transition layer sample exhibiting the highest average hardness. With the help of iron-rich phases, the samples showed enhanced tensile strength and impact absorption energy. In particular, the Fe-Ni transition layer sample demonstrated a tensile strength increase of approximately 4.8% and a 52% improvement in impact absorption energy. However, the iron-rich phase’s inherent brittleness simultaneously compromised the material′s plasticity, ultimately diminishing its elongation capacity. The elongation of the Fe-Ni transition layer was 3.1% lower than that of the sample without a transition layer.

The results show that the filling of a transition layer could improve the microstructure and mechanical properties of the material interface. However, further research into the filling method of the transition layer is still needed to achieve the best effect and the lowest-cost results.

## Figures and Tables

**Table 1:** Chemical composition of substrate and welding wire (wt. %).

Material	C	Mn	Si	S	P	Fe	Al	Ni	Cu
Q235	0.14	1.01	0.32	0.01	0.04	Balance	/	/	/
S216	/	0.014	0.03	/	/	0.88	8.84	0.005	Balance
ER120S-G	0.08	1.76	0.78	0.008	0.007	Balance	/	2.25	/

**Table 2:** Chemical composition of transition layers (wt. %).

Transition Layer	Ni	Mn	Si	Cu	Fe	Cr	Mo
Cu-Ni	30	2	3	Balance	5	4	2
Fe-Ni	30	2	4	4	Balance	4	2
Cu-Cr	/	1	2	Balance	4	20	2
Ni-Cr	Balance	1	2	/	4	22	2

## Data Availability

The original contributions presented in this study are included in the article. Further inquiries can be directed to the corresponding author.
